# Myocardial late gadolinium enhancement using delayed 3D IR-FLASH in the pediatric population: feasibility and diagnostic performance compared to single-shot PSIR-bSSFP

**DOI:** 10.1186/s12968-023-00917-0

**Published:** 2023-01-23

**Authors:** Ankavipar Saprungruang, Julien Aguet, Navjot Gill, Vivian P. Tassos, Afsaneh Amirabadi, Mike Seed, Shi-Joon Yoo, Christopher Z. Lam

**Affiliations:** 1 Division of Cardiology, Department of Pediatrics, Labatt Family Heart Centre, The Hospital for Sick Children, Toronto, ON Canada; 2 Department of Diagnostic Imaging, The Hospital for Sick Children, 555 University Avenue, Toronto, ON M5G 1X8 Canada; 3 Department of Medical Imaging, University of Toronto, Toronto, ON Canada

**Keywords:** Myocardial fibrosis, Late gadolinium enhancement, LGE, Delayed 3D IR FLASH, Single-shot PSIR-SSFP

## Abstract

**Background:**

This study compares three-dimensional (3D) high-resolution (HR) late gadolinium enhancement (LGE; 3D HR-LGE) imaging using a respiratory navigated, electrocardiographically-gated inversion recovery gradient echo sequence with conventional LGE imaging using a single-shot phase-sensitive inversion recovery (PSIR) balanced steady-state free precession (bSSFP; PSIR-bSSFP) sequence for routine clinical use in the pediatric population.

**Methods:**

Pediatric patients (0–18 years) who underwent clinical cardiovascular magnetic resonance (CMR) with both 3D HR-LGE and single-shot PSIR-bSSFP LGE between January 2018 and June 2020 were included. Image quality (0–4) and detection of LGE in the left ventricle (LV) (per 17 segments), in the right ventricle (RV) (per 3 segments), as endocardial fibroelastosis (EFE), at the hinge points, and at the papillary muscles was analyzed by two blinded readers for each sequence. Ratios of the mean signal intensity of LGE to normal myocardium (LGE:Myo) and to LV blood pool (LGE:Blood) were recorded. Data is presented as median (1st–3rd quartiles). Wilcoxon signed rank test and chi-square analyses were used as appropriate. Inter-rater agreement was analyzed using weighted κ-statistics.

**Results:**

102 patients were included with median age at CMR of 8 (1–13) years-old and 44% of exams performed under general anesthesia. LGE was detected in 55% of cases. 3D HR LGE compared to single-shot PSIR-bSSFP had longer scan time [4:30 (3:35–5:34) vs 1:11 (0:47–1:32) minutes, p < 0.001], higher image quality ratings [3 (3–4) vs 2 (2–3), p < 0.001], higher LGE:Myo [23.7 (16.9–31.2) vs 5.0 (2.9–9.0), p < 0.001], detected more segments of LGE in both the LV [4 (2–8) vs 3 (1–7), p = 0.045] and RV [1 (1–1) vs 1 (0–1), p < 0.001], and also detected more cases of LGE with 13/56 (23%) of patients with LGE only detectable by 3D HR LGE (p < 0.001). 3D HR LGE specifically detected a greater proportion of RV LGE (27/27 vs 17/27, p < 0.001), EFE (11/11 vs 5/11, p = 0.004), and papillary muscle LGE (14/15 vs 4/15, p < 0.001). Inter-rater agreement for the recorded variables ranged from 0.42 to 1.00.

**Conclusions:**

3D HR LGE achieves greater image quality and detects more LGE than conventional single-shot PSIR-bSSFP LGE imaging, and should be considered an alternative to conventional LGE sequences for routine clinical use in the pediatric population.

**Supplementary Information:**

The online version contains supplementary material available at 10.1186/s12968-023-00917-0.

## Background

Late gadolinium enhancement (LGE) cardiovascular magnetic resonance (CMR) evaluation of myocardial injury and fibrosis provides essential diagnostic and prognostic information in a variety of congenital and pediatric heart conditions [1–4]. One of the conventional methods of performing LGE imaging is by using a two-dimensional single-shot phase-sensitive inversion recovery (PSIR) balanced steady-state free precession (bSSFP; PSIR-bSSFP) sequence [1]. Recently, a three-dimensional (3D) high-resolution (HR) LGE (3D HR-LGE) option using a respiratory navigated, electrocardiographically (ECG)-gated inversion recovery gradient echo sequence has been described [5–7]. This sequence, that is widely used for contrast-enhanced CMR angiography [8, 9], has also been investigated for routine clinical use for LGE imaging in adult patients for selected conditions, such as ischemic cardiomyopathy, atrial and ventricular arrhythmias, and tetralogy of Fallot [7].

Advantages of 3D HR-LGE include higher spatial resolution, increased signal to noise, better fat saturation, and ability to perform multiplanar reconstructions. Disadvantages include a long scan time and potential for compromised quality due to inconsistent breathing, motion, significant heart rate variation, arrhythmia, or inappropriate inversion time [7]. The performance of 3D HR-LGE in the pediatric population has not yet been investigated.

The purpose of this study was to evaluate the use of a 3D HR-LGE sequence compared to a conventional single-shot PSIR-bSSFP LGE sequence for detection of LGE in routine pediatric clinical CMR.

## Materials and methods

### Study population

This single institution retrospective study received institutional Research Ethics Board approval with waiver of informed consent. Patients between 0 and 18 years of age that underwent a clinical CMR that included LGE imaging performed between January 2018 and June 2020 were included. CMRs that did not have both 3D HR-LGE and single-shot PSIR-bSSFP LGE were excluded. This was related to variable adoption of 3D HR-LGE early in the inclusion period along with standardization of our clinical practice by using conventional single-shot PSIR-bSSFP LGE exclusively as opposed to occasional alternative use of conventional segmented PSIR-FLASH LGE. Non-diagnostic exams related to technical factors were also excluded, which mostly were related to variable attempts at optimization of technique parameters early in the adoption period. Note that exams with excessive patient motion artifact were not excluded.

### CMR acquisition

Studies were performed on a 1.5 T CMR system (Avanto Fit, Siemens, Healthineers, Erlangen, Germany). Conventional LGE was performed using a two-dimensional single-shot PSIR-bSSFP sequence acquired in axial and short-axis planes 10 min following intravenous injection of 0.2 mmol/kg of gadobutrol (Gadovist; Bayer Healthcare, Berlin, Germany). 3D HR-LGE was performed using a 3D ECG-gated, respiratory-navigated inversion recovery-prepared (IR) fast low angle shot (FLASH) pulse sequence with fat saturation acquired in coronal plane, and obtained 15–20 min following intravenous administration of gadolinium-based contrast agent. Respiratory navigation was performed using pencil-beam navigation. As exact nulling of myocardium is not necessary for this sequence, an empiric inversion time of 220 ms was used with quiescent interval chosen in systole. However, when the Look-Locker acquired before conventional LGE gave a myocardial nulling inversion time ≥ 300 ms, an empiric inversion time of 280 ms was used for the 3D HR-LGE sequence with quiescent interval in diastole. Other specific sequence parameters are shown in Table 1. Isotropic axial and ventricular short axis planes in the native resolution were reconstructed for the 3D HR-LGE sequence for purposes of analysis.

**Table 1:** Technical parameters of LGE sequences

CMR parameter	Conventional LGE	3D HR-LGE
Base sequence	Single-shot PSIR-bSSFP	3D IR-FLASH
ECG gating	Mid-late diastole	Cardiac quiescence in systole or diastole
Parallel imaging × acceleration factor	GRAPPA × 2	GRAPPA × 2
Field of view	250–350 mm	250–350 mm
Pixel size	1.8 × 1.8 mm	1.1 × 1.1 to 1.4 × 1.4 mm
Slice thickness	6 mm (no gap)	1.1 to 1.4 mm
Flip angle	40˚	18˚
TR/TE	2.57/1.09 ms	330/1.4 ms
Number of averages	1–3^a^	1
Fat saturation	Yes	Yes
Acquisition plane(s)	Axial, short axis	Coronal

*ECG* electrocardiogram; *FLASH* fast low angle shot; *GRAPPA* generalized autocalibrating partial parallel acquisition; *IR* inversion recovery; *PSIR* phase-sensitive inversion recovery; *bSSFP* balanced steady-state free precession; *TE* time to echo; *TR* repetition time

^a^Smaller patients and those performed under general anesthesia were performed free breathing with more averages to reduce motion artifact and increase signal

### CMR analysis

Each sequence was analyzed by two independent, blinded readers (A.S and J.A.). Presence or absence of LGE per myocardial segment was recorded for each sequence. The left ventricle (LV) was divided into 17 myocardial segments according to standardized American Heart Association LV segmentation [10]. The right ventricle (RV) was divided into three segments: inlet, apical and outlet. In addition, presence of endocardial fibroelastosis (EFE), subendocardial LGE, mid-myocardial LGE, subepicardial LGE, or LGE at the ventricular hinge points and papillary muscles were also recorded. For cases where there was disagreement between presence and absence of LGE, consensus was reached for final analysis via a third reader (C.Z.L.).

**Fig. 1:**
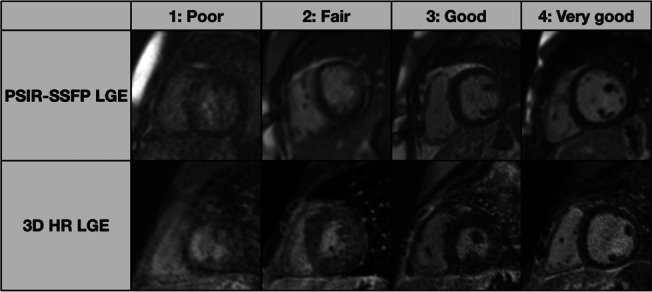
Examples of image quality ratings for conventional single-shot PSIR-bSSFP LGE and 3D HR LGE sequences. **1** poor with no clear distinction of myocardial margins; **2** fair with blurred myocardial edges; **3** good with clear myocardial margins; **4** very good with sharp myocardial margins. *3D* 3-dimensional, *HR* high-resolution, *LGE* late gadolinium enhancement, *PSIR* phase-sensitive inversion recovery, *bSSFP* balanced steady-state free precession

Image quality of each sequence was rated on a four-point ordinal scale: (1) poor with no clear distinction of myocardial margins; (2) fair with blurred myocardial edges; (3) good with clear myocardial margins; (4) very good with sharp myocardial margins (Fig. 1).

When myocardial LGE was detected on both sequences, the mean signal intensity of LGE, normal myocardium, and LV blood pool were measured using manually drawn regions of interest. LGE to myocardium (LGE:Myo) and LGE to LV blood pool (LGE:Blood) signal intensity ratios were calculated and compared.

### Statistical analysis

Continuous variables are summarized as median (1st–3rd quartiles) and categorical variables are summarized as frequency (percentage). κ-statistics were used for inter-rater agreement of LGE detection and image quality ratings between the two sequences. Wilcoxon signed rank test and chi-square analyses were used to compare conventional LGE and 3D HR LGE sequences. All statistical tests were two-sided and considered statistically significant if p < 0.05. Analyses were performed using SPSS for Windows (version 28.0.0.0, Statistical Package for the Social Sciences, International Business Machines, Inc., Armonk, New York, USA).

## Results

### Patient selection and demographics

One-hundred and sixty-six patients underwent clinical CMR that included LGE imaging during the study period. Thirty-eight patients were excluded due to the lack of concomitantly performed 3D HR-LGE and conventional single-shot PSIR-bSSFP LGE sequences. One patient was excluded due to extra-cardiac artifact obscuring the heart. Twenty-six (20%) of the remaining patients were excluded due to non-diagnostic images related to inappropriate sequence timing or inversion time, the majority as part of sequence testing and optimization early in the adoption period. This left a final study population of 102 patients (53% male; median age at CMR was 8 (1–13) years) with 45/102 (44%) of cases performed under general anesthesia. Demographic data are summarized in Table 2.

**Table 2:** Patient demographics

Characteristic	Total cases (n = 102)	Cases with LGE (n = 56)
Age (years)	8 (1–13)	10 (2–15)
Weight (kg)	26 (10–50)	39 (12–56)
Body surface area (m^2^)	0.96 (0.41–1.47)	1.21 (0.48–1.57)
Diagnosis		
Congenital heart disease	64 (63%)	41 (73%)
Cardiomyopathy	16 (16%)	9 (16%)
Systemic vasculitis	16 (16%)	5 (9%)
Systemic vasculopathy	4 (4%)	1 (2%)
Coronary anomaly	2 (2%)	0 (0%)

Data are presented as median (1st–3rd quartile) or *n* (%)

### LGE sequence quality and signal intensity ratios

**Fig. 2:**
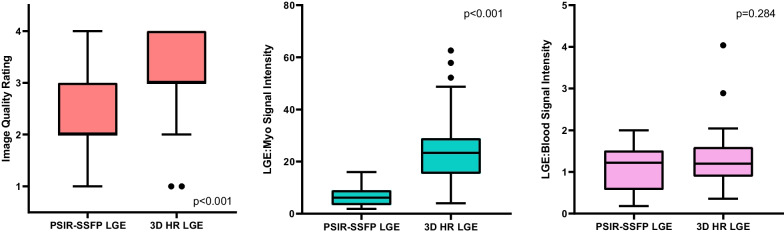
Box-and-whisker plots of image quality ratings and signal intensity ratios between conventional single-shot PSIR-bSSFP LGE and 3D HR-LGE sequences in a cohort of routine clinical pediatric patients (n = 102). Boxes show the 25th and 75th quartile ranges with median denoted by the black solid horizontal line, whiskers representing 1.5 × the quartile range, and dots representing data points beyond the whiskers

Median 3D HR-LGE scan time was 4:30 (3:35–5:34) minutes and was shorter when performed under general anesthesia [3:46 (3:01–4:26) vs 5:18 (4:31–6:17) minutes, p < 0.001]. Ninety-eight (96%) of 3D HR-LGE sequences were performed in systole. Median combined short-axis and axial single-shot PSIR-bSSFP total scan time was 1:11 (0:47–1:32) minutes and also was shorter when performed under general anesthesia [1:02 (0:42–1:23) vs 1:18 (0:54–1:35) minutes, p = 0.026]. Image quality ratings were higher for 3D HR-LGE compared with single-shot PSIR-bSSFP at 3 (3–4) vs 2 (2–3) respectively (p < 0.001). Image quality ratings were not different between sedated or awake 3D HR-LGE and PSIR-bSSFP cases (p = 0.495 and 0.468, respectively). LGE:Myo signal intensity was higher for 3D HR-LGE compared with single-shot PSIR-bSSFP at 23.7 (16.9–31.2) vs 5.0 (2.9–9.0) (p < 0.001), while there were no difference in LGE:Blood signal intensity at 1.2 (0.9–1.8) vs 1.2 (0.6–1.5) (p = 0.284). Boxplots of these data are depicted in Fig. 2.

### Detection of LGE

**Fig. 3:**
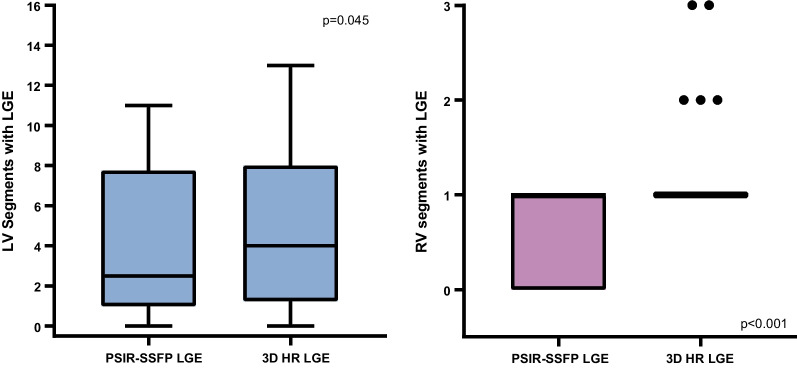
Box-and-whisker plots showing number of LV and RV segments with identified LGE on conventional single-shot PSIR-bSSFP LGE and 3D HR-LGE sequences in the cohort of pediatric patients with positive LV LGE (n = 24) and RV LGE (n = 27). Boxes show the 25th and 75th quartile ranges with median denoted by the black solid horizontal line, whiskers representing 1.5 × the quartile range, and dots representing data points beyond the whiskers. *LV* left ventricular, *RV* right ventricular

**Fig. 4:**
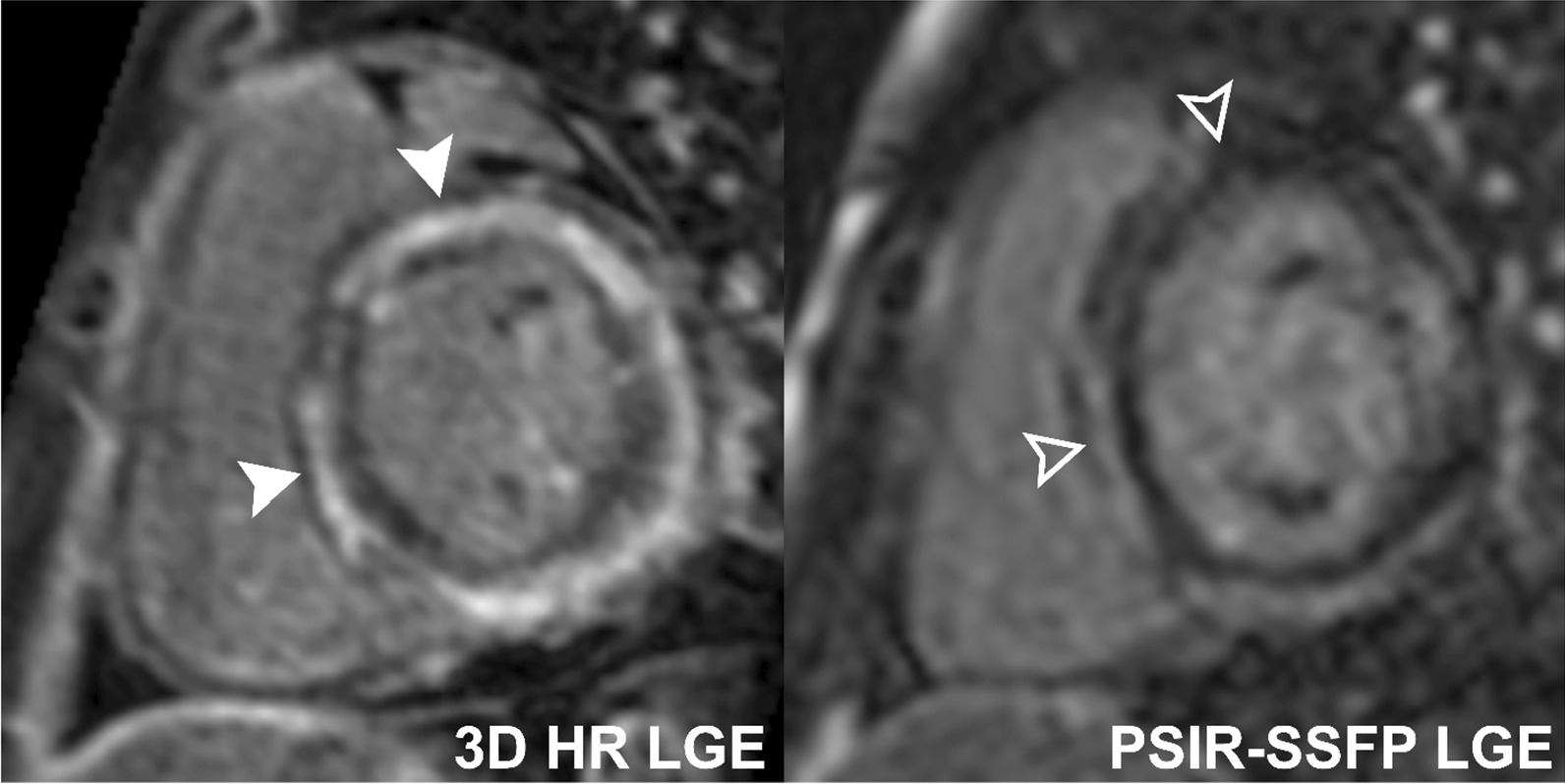
Comparison of 3D HR-LGE and conventional single-shot PSIR-bSSFP LGE sequences performed without sedation in a 14-year-old patient with myocarditis, showing patchy mid-ventricular LGE (solid and empty arrowheads). 3D HR-LGE sequences show better signal and spatial delineation of the LGE

**Fig. 5:**
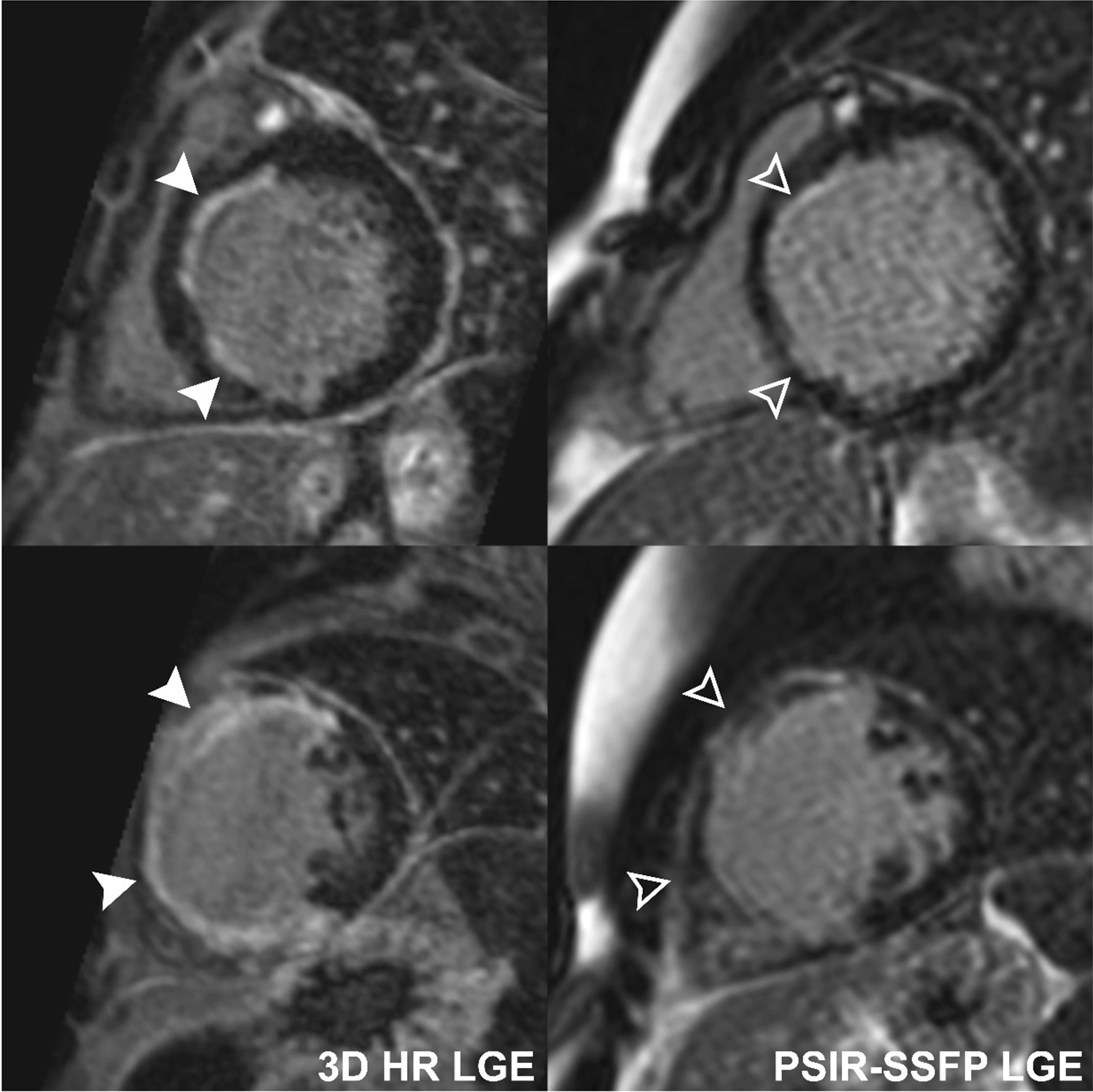
Comparison of 3D HR-LGE and conventional single-shot PSIR-bSSFP LGE sequences performed without sedation in a 15-year-old patient with Kawasaki disease, showing subendocardial LGE of the of the basal to mid left ventricle in the left main coronary artery territory (solid and empty arrowheads). 3D HR-LGE sequences show better signal and spatial delineation of the LGE

**Fig. 6:**
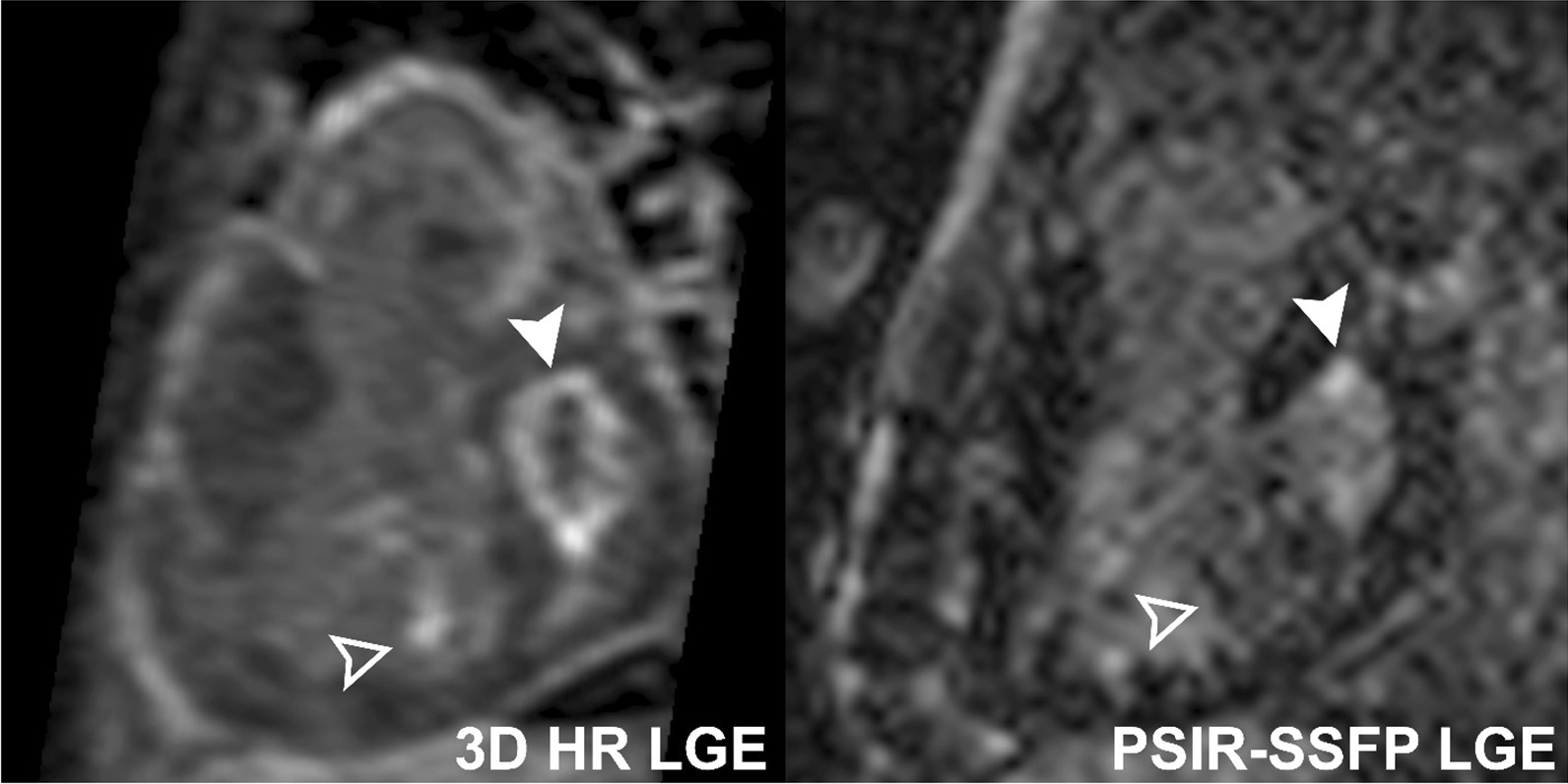
Comparison of 3D HR-LGE and conventional single-shot PSIR-bSSFP LGE sequences performed free-breathing under general anaesthesia in a 4-year-old patient with hypoplastic left heart syndrome, showing circumferential LV endocardial fibroelastosis (solid arrowheads) and focal LGE of an RV papillary muscle (empty arrowheads). Both endocardial fibroelastosis and papillary muscle LGE are not clearly identifiable on the single-shot PSIR-bSSFP sequence

**Fig. 7:**
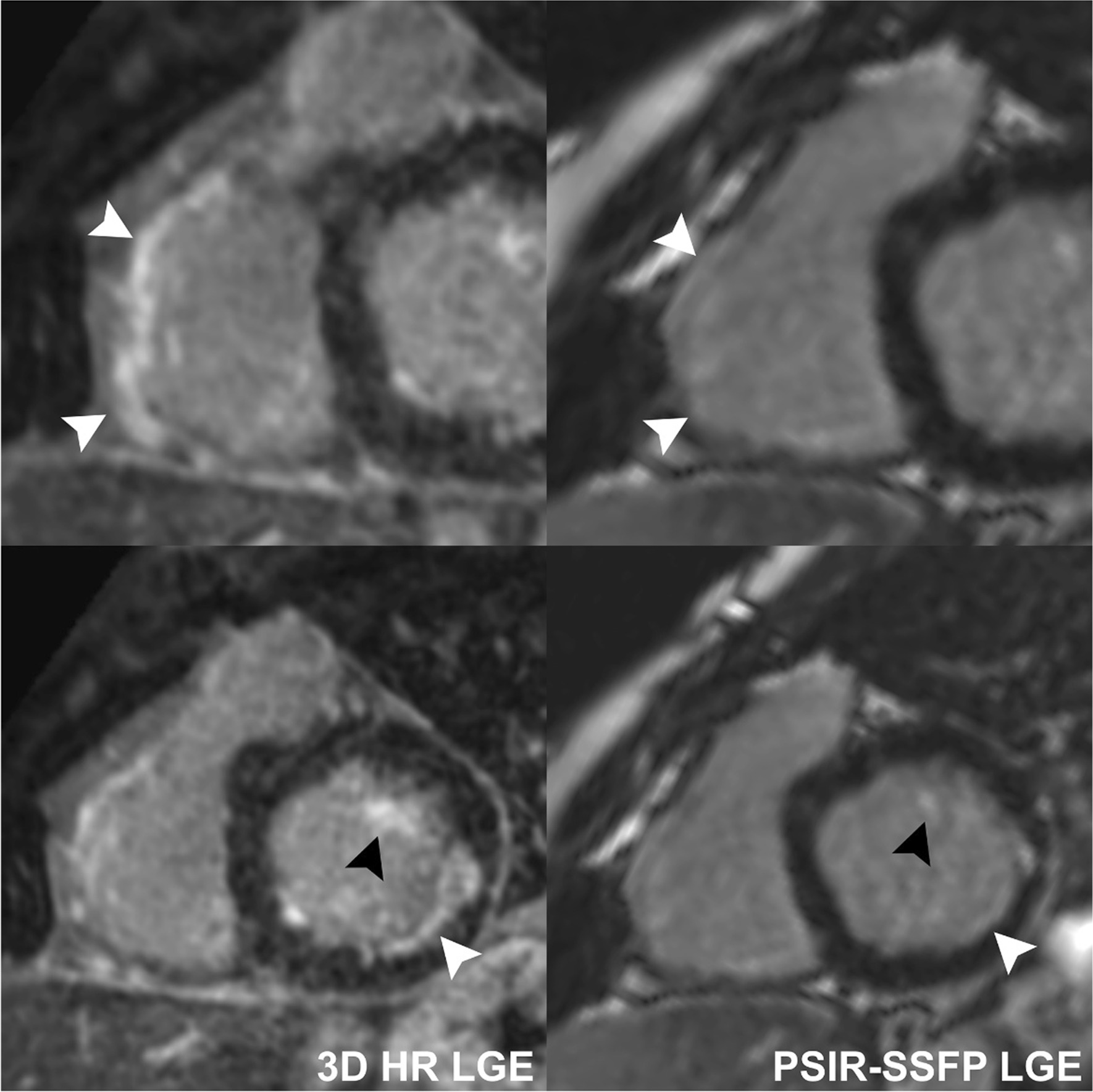
Comparison of 3D HR-LGE and conventional single-shot PSIR-bSSFP LGE sequences performed without sedation in an 11-year-old patient with Takayasu arteritis status-post left coronary artery bypass graft and right coronary artery stent, showing LGE of the basal RV free-wall and subendocardial LV free-wall (solid white arrowheads) along with LGE of the LV anterolateral papillary muscle (black arrowheads). 3D HR-LGE shows all areas of LGE better than single-shot PSIR-bSSFP

Fifty-six (55%) of exams demonstrated myocardial LGE. Specifically, 24 (24%) had LV LGE, 27 (26%) had RV LGE, 8 (8%) had LV EFE, 3 (3%) had RV EFE, 34 (33%) had subendocardial LGE, 41 (40%) had mid-myocardial LGE, 26 (25%) had subepicardial LGE, 16 (16%) had hinge point LGE, and 15 (15%) had papillary muscle LGE. The 3D HR-LGE sequence detected a greater extent of LGE than single-shot PSIR-bSSFP, with median number of positive LV segments of 4 (2–8) vs 3 (1–7) (p = 0.045), and median positive RV segments of 1 (1–1) vs 1 (0–1) (p < 0.001), respectively (Fig. 3). Moreover, LGE was detected only on the 3D HR-LGE sequence in 4/24 (17%) cases with LV LGE, 10/27 (37%) with RV LGE, 4/8 (50%) with LV EFE, 2/3 (67%) with RV EFE, 12/34 (35%) with subendocardial LGE, 12/41 (29%) with mid-myocardial LGE, 9/26 (35%) with subepicardial LGE, 6/16 (38%) with hinge point LGE, and 11/15 (73%) with papillary muscle LGE. One/24 (4%) cases with LV LGE, 1/41 (2%) with mid-myocardial LGE, 2/16 (13%) with hinge point LGE, and 1/15 (7%) with papillary muscle LGE were only seen on conventional single-shot PSIR-bSSFP LGE. Further review of these cases in conjunction with all other CMR sequences revealed that the cases of LV LGE, mid-myocardial LGE, and papillary muscle LGE were false-positives (these were left as positive cases for purposes of statistical analysis). This data are summarized in Table 3. Representative side-by-side comparison examples are depicted in Figs. 4, 5, 6, 7.

**Table 3:** Detection of LGE using 3D HR-LGE and conventional single-shot PSIR-bSSFP LGE sequences in 56 patients with positive LGE

Location of LGE	LGE on 3D HR- LGE only (%)	LGE on PSIR-bSSFP LGE only (%)	*p*-value
LGE in any location (n = 56)	13 (23)	0 (0)	< 0.001
LV LGE (n = 24)	4 (17)	1 (4)^a^	0.346
RV LGE (n = 27)	10 (37)	0 (0)	< 0.001
LV or RV EFE (n = 11)	6 (55)	0 (0)	0.004
Subendocardial LGE (n = 34)	12 (35)	0 (0)	< 0.001
Mid-myocardial LGE (n = 41)	12 (29)	1 (2)^a^	0.002
Subepicardial LGE (n = 26)	9 (35)	0 (0)	0.002
Hinge point LGE (n = 16)	6 (38)	2 (13)	0.220
Papillary muscle LGE (n = 15)	11 (73)	1 (7)^a^	< 0.001

^a^False positives after re-review

*3D* 3-dimensional, *EFE* endocardial fibroelastosis, *HR* high resolution, *LGE* late gadolinium enhancement, *LV* left ventricle, *PSIR* phase-sensitive inversion recovery, *R* right ventricle, *bSSFP* balanced steady-state free precession

### Inter-rater agreement

Inter-rater agreement via weighted κ-statistics was 0.86 [95% CI 0.79–0.93] for 3D HR LGE image quality ratings, 0.60 [95% CI 0.46–0.74] for single-shot PSIR-bSSFP LGE image quality ratings, and ranged from 0.42 to 1.00 for detection of LGE depending on myocardial location and sequence. Inter-rater agreement was not clearly different between the two techniques, with overlapping 95% confidence intervals. This data is summarized in Additional file 1: Table S1.

## Discussion

In this study, a 3D HR-LGE sequence was compared with conventional single-shot PSIR-bSSFP LGE imaging as a clinical routine in the pediatric population. The key findings of this work are that 3D HR-LGE is feasible for routine use in pediatric clinical CMR, and compared to single-shot PSIR-bSSFP LGE, achieves greater image quality and detects more LGE, particularly for the RV, EFE, and papillary muscles.

Some of the disadvantages of a 3D HR-LGE sequence relate to the long scan time that may result in patient motion and suboptimal image quality. In this cohort, 3D HR-LGE was performed with a median scan time of 4.5 min, which is shorter than reported in adults with an average of 10 min [7]. This may be related to faster heart rates, faster respiratory rates, and smaller size of children. Conversely, single-shot PSIR-bSSFP sequences suffer from low signal in small children with fast heart rates. 3D HR-LGE may thus be well suited for the pediatric population, particularly when the study is performed under general anesthesia or sedation where respirations are better controlled. Indeed, in this study we confirm that 3D HR-LGE shows greater image quality and a four to fivefold higher LGE:myo signal intensity compared with conventional LGE.

This translated to superior diagnostic performance with 3D HR-LGE detecting both more patients with LGE, and greater extent of LGE when present. In fact, in 23% of patients that had LGE, the LGE was not detectable by single-shot PSIR-bSSFP. The performance of 3D HR-LGE was particularly superior for LGE of small or thin structures, comparable to the use of 3D HR-LGE for the atrium in adults [11]. In our population, this improved performance was most evident for RV LGE, EFE, and papillary muscle LGE, where single-shot PSIR-bSSFP missed up to 37% of RV LGE, 55% of EFE, and 38% of papillary muscle LGE. Some of the superior performance for RV LGE may be related to the fact 3D HR-LGE was primarily performed in systole whereas single-shot PSIR-bSSFP was performed in diastole, however, the improved spatial resolution and signal of the 3D HR-LGE sequence was also likely beneficial. The fact that 3D HR-LGE was superior to PSIR-bSSFP for detection of LGE in all myocardial layers, along with the similar LGE:blood signal intensity, suggests that improved contrast between subendocardial LGE and blood pool was not necessarily the primary driver of superior performance in this study. Therefore, this technique may be adjunctive to proposed dark-blood or gray-blood LGE strategies that aim to optimize LGE:blood contrast [12]. Importantly, 3D HR-LGE did not miss any clinically significant LGE in our study (only two cases of hinge point LGE that was related to patient motion on the 3D HR-LGE sequence), and in fact, helped to show apparent LGE on single-shot PSIR-bSSFP sequences were false positives in 2 cases. Our data therefore supports using 3D HR-LGE as a clinical standard in the pediatric population.

Of practical considerations for implementation, this sequence is widely available and can be repeated for both angiography and LGE imaging in the same exam. It does not preclude perfusion imaging, conventional LGE, or T1/ECV mapping. At our institution we no longer perform conventional LGE sequences for non-cardiomyopathy patients.

## Limitations

Our study is limited by its retrospective design using clinical cases that did not allow for strict control of technical parameters. The true failure rate of the 3D HR-LGE sequence could not be determined as we included examinations performed during our testing and adoption period, which produced some non-diagnostic exams due to varying of CMR parameters. However, with subsequent experience and optimization, recent internal quality improvement efforts analyzing 100 consecutive patients showed that our current non-diagnostic rate is only 4/100 (4%), 3 related to excessive patient motion (poor patient cooperation or inconsistent breathing pattern) and 1 related to technical artifact. Moreover, our results may be confounded by heterogeneity in exams including comparisons of 3D HR-LGE acquisitions in systole versus single-shot PSIR-bSSFP in diastole. Nonetheless, we feel our results remain valid, showing that 3D HR-LGE as we performed in clinical routine is superior to the conventional method of acquiring single-shot PSIR-bSSFP. Finally, this study was limited by the lack of pathologic correlation or gold-standard for presence of LGE.

## Conclusion

3D HR-LGE is feasible for routine use in pediatric clinical CMR, and compared to single-shot PSIR-bSSFP LGE, achieves greater image quality and detects more LGE. Given wide-spread availability and superior performance, 3D HR-LGE can be used as the primary standard LGE sequence in the pediatric population, with conventional LGE sequences reserved only for situations when proper ECG gating and/or respiration navigation are not possible.

## Supplementary Information

**Additional file 1:** **Table S1.** Inter-rater agreement for presence or absence of LGE by myocardial location for conventional single-shot PSIR-SSFP LGE and 3D HR LGE sequences.

## Data Availability

The datasets used and/or analysed during the current study are available from the corresponding author on reasonable request.

## Abbreviations

bSSFP: Balanced steady-state free precession
CHD: Congenital heart disease
CMR: Cardiovascular magnetic resonance
ECG: Electrocardiogram
EFE: Endocardial fibroelastosis
FLASH: Fast low angle shot
FOV: Field of view
GBCA: Gadolinium-based contrast agent
GRE: Gradient echo
HR: High resolution
IR: Inversion recovery
LGE: Late gadolinium enhancement
LV: Left ventricle/left ventricular
PSIR: Phase-sensitive inversion recovery
RV: Right ventricle/right ventricular
TE: Echo time
TI: Inversion time
TR: Repetition time
