# Supplementary material for: Myocardial late gadolinium enhancement using delayed 3D IR-FLASH in the pediatric population: feasibility and diagnostic performance compared to single-shot PSIR-bSSFP
Source: J Cardiovasc Magn Reson. 2023 Jan 23;25:2. doi: 10.1186/s12968-023-00917-0 (PMC9869523; doi:10.1186/s12968-023-00917-0)
Supplement: Supplementary file 1 — Additional file 1: Table S1. Inter-rater agreement for presence or absence of LGE by myocardial location for conventional single-shot PSIR-SSFP LGE and 3D HR LGE sequences. [file 12968_2023_917_MOESM1_ESM.docx]

**Table S1.** Inter-rater agreement for presence or absence of LGE by myocardial location for conventional single-shot PSIR-SSFP LGE and 3D HR LGE sequences.

| **Location** | **κ-value [95% CI]** | |
| --- | --- | --- |
|  | **PSIR-bSSFP LGE** | **3D HR-LGE** |
| **LV LGE** | 0.92 [0.84-1.00] | 0.91 [0.84-0.98] |
| **RV LGE** | 0.47 [0.25-0.70] | 0.70 [0.44-0.96] |
| **RV EFE** | 1.00 [1.00-1.00] | 0.56 [0.11-1.00] |
| **LV EFE** | 0.80 [0.40-1.19] | 0.78 [0.55-1.02] |
| **Subendocardial LGE** | 0.67 [0.48-0.85] | 0.76 [0.61-0.90] |
| **Mid-myocardial LGE** | 0.66 [0.49-0.83] | 0.77 [0.64-0.91] |
| **Subepicardial LGE** | 0.42 [0.12-0.71] | 0.60 [0.39-0.80] |
| **Hinge point LGE** | 1.00 [1.00-1.00] | 0.96 [0.87-1.04] |
| **Papillary muscle LGE** | 0.74 [0.40-1.08] | 0.85 [0.69-1.02] |

*CI = confidence interval, EFE = endocardial fibroelastosis, LGE = late gadolinium enhancement, LV = left ventricle, PSIR = phase-sensitive inversion recovery, RV = right ventricle, bSSFP = balanced steady-state free precession*
